# Belgrave Hospital for Children

**Published:** 1903-12-05

**Authors:** 


### BELGRAVE HOSPITAL FOR CHILDREN.

On Tuesday, December 1st, the inauguration of the work of this hospital, which had been opened by H R H. Princess Henry of Battenberg last July, took place under the auspices of the Bishop of Rochester.

The large hall was quite filled with guests when the Bishop entered, and, after conducting a short service of dedication, made a short speech. Having alluded to the opening ceremony in July, he said that the present ceremony was of a more domestic character, and the gathering consisted of those who were more closely interested with the building, the workers themselves and their immediate neighbours. The building of this hospital was an instance of what was likely to be done on a larger scale in the future, namely, the provision of hospital accommodation on the south side of the Thames. This hospital was new here, but old elsewhere, it having been first established in Pimlico nearly forty years ago. He chiefly associated with that change the name of the late Duke of Westminster, who in his position was one of the first citizens of the West End of London to recognise the obligations of the West End to this home of labour on the south side of the river. What the late Duke personally recognised in a liberal and practical way was now being recognised by public opinion. The moving of this hospital was, they had a right to hope confidently, the beginning of better things, for they had now heard of the prospective moving of King's College Hospital, a step which he was heartily glad had been decided upon. With regard to the Belgrave Hospital, it was not yet complete, although £40,000 had been spent upon it. Now that they had received this noble contribution to their local life, he trusted that they would all rally round it in every way possible. The good that was done by hospitals was not confined to the actual patients treated in them but percolated throughout the whole neighbourhood. He could not promise that the promoters of the hospital would meet with much return in the way of money, for they were a very poor lot down there, but they would find a real return in gratitude and interest. He wished the hospital godspeed with all his heart and considered it a privilege to have been able to do anything in its support. Before closing his remarks he made an appeal for the right and proper provision for the spiritual needs of the nurses of the hospital. He trusted that if it was felt that they needed a chapel within the building as in other hospitals such as Guy's, that this need would be supplied by the authorities. In some of the foreign hospitals, owing to the prevalence of the non-religious and anti-religious spirit, there was no provision for the spiritual needs of the patients or the nurses who had so heavy a strain upon them. As yet this was not the case in England.

Mr. W. H. Warner, vice-chairman of the hospital, made an appeal for help towards the funds of the hospital to enable them to carry on their work.

The hospital was first opened for work on that day, and admitted two in-patients and treated 14 out-patients.

